# Emotional Arousal at Memory Encoding Enhanced P300 in the Concealed Information Test

**DOI:** 10.3389/fpsyg.2017.02334

**Published:** 2018-01-10

**Authors:** Akemi Osugi, Hideki Ohira

**Affiliations:** ^1^Forensic Science Laboratory, Kobe, Japan; ^2^Department of Psychology, Graduate School of Environmental Studies, Nagoya University, Nagoya, Japan

**Keywords:** emotional arousal, Concealed Information Test, event-related potential, P300, mock crime

## Abstract

Previous studies have reported that the concealed information test (CIT) is a reliable and powerful method for detecting information. However, the external validity of the CIT studies has not been fully proven. In particular, few studies have examined the effects of emotional arousal at memory encoding on physiological responses in the CIT. The present study investigated the influence on the CIT of the magnitude of emotional arousal at memory encoding of a mock crime, using the P300 component of the event-related brain potential (ERP). In accord with the assumptions of excitation-transfer theory, we presented emotionally arousing pictures before a mock crime. Participants were randomly assigned to either a high emotional arousal group (*n* = 10) or a low emotional arousal group (*n* = 11), viewing pictures expected to arouse emotion at a high or low level, respectively. Subsequently, all participants enacted the same mock crime, in which they were instructed to stab a pillow with a sharp-edged tool (e.g., a kitchen knife or ice pick) as if harassing a mannequin lying on a bed. After the antecedent emotional experience, the P300-based CIT was conducted. Participants in the high arousal group showed significantly greater P300 amplitudes in response to a probe stimulus compared with the low arousal group. No differences were found between the groups in response to irrelevant stimuli. These results support the notion that emotional arousal influences the P300 in the CIT paradigm.

## Introduction

The concealed information test (CIT) is a method of detecting information strongly related to an examinee’s memory (Verschuere et al., 2011a). The CIT has been established as a reliable and powerful method for detecting information in numerous studies (for reviews, see Ben-Shakhar, 2012; Meijer et al., 2014). Although the CIT has been applied in the practical forensic field in several countries, including Japan (Nakayama, 2002; Osugi, 2011; Ogawa et al., 2015; Zaitsu, 2016), the external validity of the CIT studies has been an open question among researchers because it is difficult to obtain useful field data. Some studies have compared the results of laboratory experiments with those of field examinations (Osugi, 2010, 2018; Zaitsu, 2016), but the external validity of the CIT studies has not yet been established.

Emotional arousal is one of potential factors that seems to differ between laboratory experiments and field situations. Although emotional arousal during retrieval in the CIT has been investigated in several studies (Kugelmass and Lieblich, 1966; Bradley and Janisse, 1981; Verschuere et al., 2011b), little empirical attention has focused on the role of emotional arousal at memory encoding in the CIT. Studies in the past several decades have provided considerable evidence suggesting that emotional arousal strongly influences memory (for a review, see Christianson, 1992; Hamann, 2001; McGaugh, 2004). Using emotionally arousing stimuli, some studies have investigated the effect of emotional arousal and reported physiological responses during encoding; They obtained larger skin conductance responses (SCR) (Bradley et al., 1992), larger event-related brain potentials (ERP) (Palomba et al., 1997; Dolcos and Cabeza, 2002), and higher activation of amygdala (Hamann et al., 1999; Dolcos et al., 2003, 2004) to emotionally arousing stimuli than to low-arousing or neutral stimuli. These studies also reported better memory performance in the subsequent memory test (e.g., recall test and recognition test), that is, emotionally arousing stimuli were remembered and retrieved better than low-arousing or neutral stimuli, even after long interval (Dolcos et al., 2005). Regarding memory storage, numerous eyewitness memory studies have revealed that people tend to have good retention of detailed information from highly emotionally arousing events (Christianson, 1992). Neuroimaging studies have also demonstrated that emotional arousal enhances memory consolidation (Hamann, 2001; McGaugh, 2004). Taken together, emotional arousal seems to strongly affect memory, interacting in various memory stages.

Although it may seem natural that emotional arousal at memory encoding would influence the detection of crime-related information by the CIT, few previous studies have examined this. There have been two CIT studies focusing on emotional arousal. Peth et al. (2012) manipulated emotional arousal with a confederate who unexpectedly entered the storeroom in which the participants were enacting the mock crime. Half of the guilty participants underwent the induction of arousal during the mock crime task, and autonomic responses of all participants were measured in the CIT. This study reported no significant differences in emotional arousal on the physiological responses between groups during the CIT. Klein Selle et al. (2017) also manipulated emotional arousal by showing a police case-file including emotionally arousing pictures and neutral pictures with a description of the crime in their study phase. They used the same pictures as the probe in the CIT, and found the detection efficiency on the SCR was better in the emotional stimuli condition compared with the neutral stimuli condition.

The results from these studies seem inconsistent, but these experiments highlight three issues for further examination. The first relates to the manipulation of emotional arousal by Peth et al. (2012). In their experiment, the arousal manipulation was a brief interruption made by a confederate. Although the intention was to induce emotional arousal, the manipulation may have introduced other confounding factors. For example, participants’ attention may have been affected by the interruption. Moreover, participants’ concentration on the mock crime task may have been affected. Either of these possibilities could have influenced memory encoding for the probe item, which was the focus of questioning in the CIT. In addition, this manipulation was conducted only for the arousal induction group, not for the no-arousal group. Although the interruption may only have influenced the arousal induction group, its influence may have been contaminated by other factors that are relevant to CIT performance. The results indicated that participants in the arousal induction group were not able to recognize as many probe stimuli as were participants who were not disturbed during the mock crime. To resolve these potential issues, a new method of emotional arousal manipulation is needed.

In the current study, we separated the manipulation of emotional arousal from the mock crime task by presenting emotionally arousing pictures before the crime task. Pictures were selected from the International Affective Picture System (IAPS; Lang et al., 1999), and all participants enacted the same mock crime after the manipulation. According to excitation-transfer theory (Zillmann, 1978, 1979), which emphasizes the effect of arousal in emotional transfer, a temporally close emotion-eliciting event amplifies and energizes the experience of subsequent emotions, causing them to be felt more intensely. Based on this theory, we assumed that the experience of viewing high or low emotionally arousing pictures (the manipulation of emotional arousal) would affect the intensity of emotional arousal while acting as a mock criminal (performing the mock crime task) differently. This manipulation allowed us to investigate the influence of emotional arousal directly, without introducing differences in the action or other factors of the mock crime task. Although the action involved in the mock crime used in the previous studies has typically involved stealing an object (e.g., Carmel et al., 2003; Nahari and Ben-Shakhar, 2011; Peth et al., 2012; see meta-analysis, Meijer et al., 2014), we adopted a new type of action in the present study: participants were instructed to enter a room in which a female mannequin was lying on a bed, and perform a crime of intimidation – stabbing the pillow near the mannequin’s head with a sharp tool – to ensure that the probe was strongly encoded.

The second issue is raised by Klein Selle et al.’s (2017) study. Although they found an effect of emotional arousal on detection efficiency in the CIT, we suggest that their effect seemed to reflect the emotional arousal at encoding and at retrieval because they used the same emotional arousing stimuli not only at encoding but also in the CIT. In contrast, neutral stimuli were used consistently both at encoding and in the CIT in their neutral condition. To investigate the effect of emotional arousal at memory encoding without contamination of the emotional effect during the CIT (at retrieval), neutral stimuli have to be presented in the CIT for both the emotional arousing condition and the non-arousing condition. Thus, in this study, we used the same neutral pictures of a sharp tool as stimuli for both the High Arousal group and the Low Arousal group. It should be noted that we manipulated state arousal using affective pictures just before the mock crime, but did not use the affective pictures in the CIT. We used neutral sharp tool pictures as stimuli for the CIT because we focused on the effect of state arousal during the mock crime, not on the effect of affective pictures in the CIT.

The last issue raised by both studies (Peth et al., 2012; Klein Selle et al., 2017) is related to the physiological indices used. Because different findings were reported in these studies, the effect of emotional arousal on autonomic indices in the CIT studies remains controversial. However, several previous studies have reported a relationship between the P300 component of the ERP, and emotional arousal (for a review, see Olofsson et al., 2008). Several studies also investigated the relationship between memory and emotional arousal using the P300, reporting that high-arousal pictures elicited greater P300 responses than neutral or low-arousal pictures at encoding (Palomba et al., 1997; Dolcos and Cabeza, 2002) and at recollection (Xu et al., 2015). These studies have established that an effect of emotional arousal can be found using the P300 in the CIT. Therefore, we adopted the P300 in the present study. In previous studies of the CIT, P300 amplitude has been utilized to detect critical details of a crime or other concealed information, commonly reporting that the critical item, the probe, elicited larger P300 responses than non-critical items, called irrelevant stimuli (e.g., Farwell and Donchin, 1991; Johnson and Rosenfeld, 1992; Allen and Iacono, 1997; Rosenfeld et al., 2004; Kubo and Nittono, 2009; Gamer and Berti, 2012; Matsuda et al., 2013a).

In the current study, we investigated the effects of emotional arousal at memory encoding in the P300-based CIT paradigm, manipulating participants’ emotional state beforehand, and using an arousing mock crime. We hypothesized that there would be a significant difference between responses to the probe and responses to the irrelevant stimuli regardless of the emotional arousal condition. We also hypothesized that P300 amplitudes elicited by the probe would differ by group, whereas those elicited by the irrelevant stimuli would not differ by group: we predicted that the High Arousal group would experience stronger emotional arousal during the mock crime and would therefore elicit greater P300 amplitudes to the probe compared with the Low Arousal group, which would experience a lower level of emotional arousal. Thus, we hypothesized that detection of the probe would be easier in the High Arousal group than in the Low Arousal group.

## Materials and Methods

### Participants

Twenty-four undergraduates (12 male, 12 female) voluntarily participated in this experiment. Their mean age was 21.21 years (range 19–26). All were right-handed, had normal or corrected vision, and had no self-reported history of neurological disease. Participants were randomly assigned to either a High Arousal group or a Low Arousal group. This experiment was conducted in accordance with the ethical principles of Declaration of Helsinki, and we followed the necessary procedures. All participants gave informed written consent to participate in the study. The study was reviewed and approved by the Ethics Committee of the Graduate School of Environmental Studies, Nagoya University, Japan.

### Manipulation of Emotional Arousal

The IAPS (Lang et al., 1999), a standardized collection of color pictures that arouse emotion and that have been rated by large groups of North American participants in terms of valence, dominance, and arousal, was used to manipulate participants’ emotional arousal. From the IAPS, ten high emotionally arousing pictures (High Arousal pictures) and ten low emotionally arousing pictures (Low Arousal pictures)^1^ were selected such that the mean arousal scores were significantly greater in the high emotionally arousing pictures condition than in the low emotionally arousing pictures condition [*t*(9) = 12.573, *p* < 0.001, *d* = 4.85]^2^. The mean valence scores were not significantly different between high and low emotionally arousing pictures [*t*(9) = -0.935, *p* = 0.366, *d* = -0.32], although in this study, regardless of condition, all pictures have negative valence. The mean scores differed in the dominance dimension [*t*(9) = -2.444, *p* = 0.037, *d* = -1.21]. Arousal and valence are the most important dimensions for this manipulation, because they are considered to capture the global and basic elements of emotion. Thus, it is likely that the difference in the dominance dimension has little influence on this manipulation. All pictures were unrelated to the mock crime task in this study, and were used only to manipulate emotional arousal before the mock crime task. In this manipulation, each picture was projected for 10 s on a CRT display situated 1 m in front of the participants’ eyes. High Arousal pictures were presented to the High Arousal group, and Low Arousal pictures were presented to the Low Arousal group. Participants were instructed that 10 pictures would be presented and that they should attend to each picture for the entire time it appeared on the screen.

### Mock Crime

All participants were asked to choose one of five envelopes. Two keywords were inserted into each envelope. After choosing an envelope, participants were asked to spend 1 min memorizing the two keywords inside. Keyword 1 was the name of a sharp tool that was supposed to have been used in the mock crime (choices were kitchen knife, box-cutter, ice pick, sickle, and saw). Keyword 2 was “pillow,” regardless of which envelope participants chose. Participants in both emotional arousal groups were instructed to stab the object represented by Keyword 2 with the object represented by Keyword 1. Unbeknownst to the participants, Keyword 1 items were counterbalanced. After choosing an envelope and engaging in the 1-min memorization period, all participants performed a recall test, in which they wrote the two keywords five times to confirm and enhance their memory. All participants then proceeded to the mock crime phase. They were instructed to move to a separate room by themselves and to look for the item indicated by Keyword 1 in that room. After finding the item, they were instructed to stab Keyword 2 (pillow) with the Keyword 1 item a few times, as forcefully as possible, as if intimidating an adult female represented by a mannequin that had been laid on a bed with its head on the pillow. Thus, participants were instructed to stab the pillow very close to the mannequin’s head. Participants were also instructed to remain in the room for more than 10 min, and to smuggle the Keyword 1 item out of the room when they left, keeping the item in a bag they had been given, carefully covered with a towel.

### CIT

The P300-based CIT was then administered. Participants were told that the experiment was designed to check whether they had information about Keyword 1, the tool that was used in the prior mock crime. They were also instructed to pretend to be innocent and to make an effort to avoid positive detection by electroencephalogram (EEG). As a motivational incentive, the participants were told that if they were not detected, they would receive a monetary reward. Before the actual CIT, participants took part in a card test to ensure they understood the trial timing and became accustomed to it. The card test is typically used for all examinees in the field in Japan, to confirm their understanding of the procedure and assess their physiological response patterns, as well as to check the apparatus (Osugi, 2011). In the card test participants first chose one of five cards, and memorized the number written on the card. They were instructed to press the left button of a computer mouse when they saw the number 1, as a target, and the right button of the mouse when they saw other numbers, including a number that had been chosen by the participants, which denoted the probe, and four irrelevant numbers. The practice session consisted of 60 trials. In the actual CIT, rather than asking “Did you use this tool in the mock crime?,” six pictures of the sharp-edged tool, as described in detail below, were presented at a constant interval. Participants were required to press the response button with their right hand as quickly as possible when they recognized the stimulus, instead of saying “No.” To ensure that participants attended to the stimuli and performed the stimulus classification prerequisite for the elicitation of the P300, they were asked to press the left button of the mouse only when they recognized the picture of scissors, which served as a target stimulus. All pictures used in the CIT were presented separately for participants in this part of the experiment. Participants were required to carefully survey all pictures and to learn the picture of scissors as the target stimulus. They were also instructed to press the right button of the mouse when any of five other pictures were presented; these pictures included a probe and four irrelevant stimuli. The probe was the picture of the tool the participant had used in the mock crime, and the four irrelevant stimuli were unrelated to the mock crime.

#### Stimulus Presentation

**FIGURE 1:**
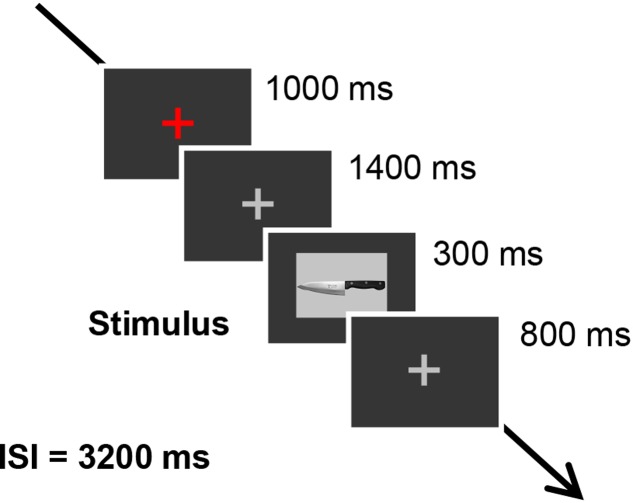
Trial timing of stimulus presentations.

The six previously described pictures of sharp tools were used as stimuli. The scissors were always used as the target stimulus, and the other pictures, which were kitchen knife, box-cutter, ice pick, sickle, and saw, were used as probe stimulus or irrelevant stimuli. The pictures were all 12.9 cm by 9.4 cm and were projected on a CRT display situated 1 m in front of the participants. Each trial began with a red fixation cross, presented for 1000 ms in the middle of the screen, followed by a gray fixation cross presented for 1400 ms. Subsequently, each sharp tool picture was presented for 300 ms, with the gray fixation cross appearing for 800 ms between each tool picture. The inter-stimulus interval was 3200 ms. The trial timing was shown in **Figure 1**. Participants were told not to blink when the sharp tool pictures and the gray fixation cross were presented. We used Presentation software (Neurobehavioral Systems, Inc., Albany, CA, United States) to control the stimulus presentation. Each tool picture was presented 20 times in a session in random order, and there were three sessions in total. Between sessions, participants took a 2-min break.

#### Physiological Responses

The EEG was recorded from locations Fz, Cz, and Pz, according to the International 10/20 System. The reference electrode was placed on the nose, and the forehead was grounded. Electrooculograms (EOG) were also recorded from electrodes placed supra-orbitally to the left eye. Ag/AgCl electrodes were used, and electrode impedance did not exceed 5 kΩ. Signals were amplified with an MP100 system (BIOPAC Systems, Inc., Goleta, CA, United States) with a 35 Hz low pass filter and 0.1 Hz high pass filter. Amplified EEG and EOG signals were digitized at a rate of 1000 points per second and recorded by EPLYZER II (Kissei Comtec Co., Ltd., Matsumoto, Nagano, Japan). After recording, the EEG in the 1100-ms period between 100 ms prior to stimulus onset to 1000 ms after stimulus onset was analyzed with EPLYZER II (Kissei Comtec Co., Ltd., Matsumoto, Nagano, Japan). The average amplitude of the 200-ms pre-stimulus interval was used as the baseline. In the averaging procedure, epochs in which the signal amplitudes exceeded ±100 μV on any of the electrodes were removed by visual inspection. Only epochs with behavioral responses in the range of 200–800 ms were averaged with respect to each stimulus type: target, probe, and irrelevant. We used at least 30 artifact-free epochs per stimulus (one target, one probe, four irrelevant stimuli each) because Cohen and Polich (1997) reported that P300 amplitudes become statistically stable after 20 target trials are obtained, and change very little with 30 or more target trials.

### Self-Report

The Japanese version of the UWIST Mood Adjective Checklist, short version (JUMACL; Shirasawa et al., 1999) was used in this study. The JUMACL comprises two subscales of 10 items each: Energetic Arousal (ranging from feeling sleepy to feeling awake) and Tense Arousal (ranging from feeling calm to feeling nervous). These subscales are sensitive to external stressors, and participants scoring high on Energetic Arousal tend to report feeling vigorous, bright, and active, while high Tense Arousal scores imply nervousness, jitters, and tenseness. The participants were instructed to rate the applicability of each adjective to their present mood using a four-choice symmetric format, as “definitely,” “slightly,” “slightly not,” or “definitely not.” Responses were scored from 4 for “definitely” to 1 for “definitely not.”

### Procedure

All participants were informed before starting the experiment that they would be asked to enact a mock crime and take the CIT, which involved ERP measurement. Informed consent was obtained from all participants. After general instructions had been given, the manipulation of emotional arousal was conducted for each group. All participants then enacted the mock crime. After physiological recording equipment had been attached, the CIT was conducted. All participants were required to give subjective ratings of their emotional arousal using the JUMACL at three time points, before the manipulation of emotional arousal (before IAPS), after the manipulation of emotional arousal (after IAPS) and after the mock crime (after crime). At the end of the experiment, participants were asked how they had felt during the mock crime and the CIT, and their memory of the probe was confirmed with a short questionnaire.

## Results

Repeated measures analyses of variance (ANOVA) were conducted for JUMACL scores, reaction times (RT) and P300 amplitudes. The Greenhouse-Geisser correction was used to account for violation of sphericity, which is likely when repeated measures factors have more than two levels. The Bonferroni correction was used for *post hoc* comparisons in all cases, and effect sizes in ANOVA were shown using partial eta squared ($\eta_{p}^{2}$). These calculations were performed with PASW Statistics 18 (IBM). Data from three participants were discarded because of excessive electrooculographic artifacts in recording of ERPs, leaving a final sample of 21 participants (High Arousal group; *n* = 10, Low Arousal group; *n* = 11).

### Manipulation Check

**FIGURE 2:**
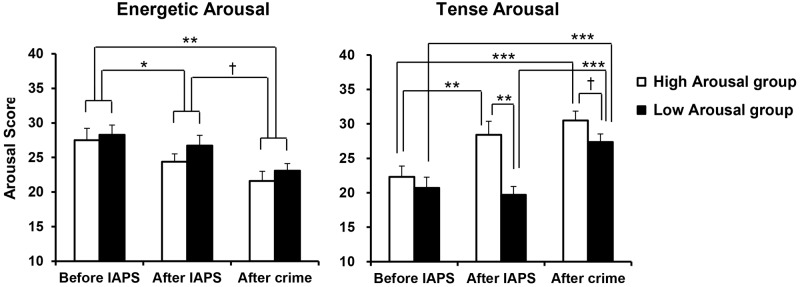
Mean scores for Energetic Arousal and Tense arousal, by group. Error bars indicate standard errors. ^†^*p* < 0.10, ^∗^*p* < 0.05, ^∗∗^*p* < 0.01, ^∗∗∗^*p* < 0.001.

The mean scores on Energetic Arousal and Tense Arousal are shown in **Figure 2**. For each arousal scale, a GROUP (High, Low) × PERIOD (before IAPS, after IAPS, after crime) ANOVA was conducted. First, for Energetic Arousal, there was a significant main effect of PERIOD [*F*(2,38) = 12.116, *p* < 0.001, $\eta_{p}^{2}$ = 0.389]. *Post hoc* comparisons showed that Energetic Arousal was significantly lower after crime than before IAPS (*p* = 0.001) and also significantly lower after IAPS than before IAPS (*p* = 0.039) regardless of Emotion group. In addition, Energetic Arousal demonstrated a marginally significant decrease after crime compared with after IAPS (*p* = 0.063). For Tense Arousal, a significant main effect of PERIOD [*F*(2,38) = 24.262, *p* < 0.001, $\eta_{p}^{2}$ = 0.561], a significant main effect of GROUP [*F*(1,19) = 7.024, *p* = 0.016, $\eta_{p}^{2}$ = 0.270] and a significant interaction of GROUP × PERIOD [*F*(2,38) = 5.943, *p* = 0.006, $\eta_{p}^{2}$ = 0.238] were observed. According to *post hoc* comparisons, Tense Arousal in the High Arousal group was significantly heightened after IAPS compared with before IAPS (*p* = 0.002), and there was no difference between after IAPS and after crime. However, Tense Arousal in the Low Arousal group was significantly heightened after crime compared with after IAPS (*p* < 0.001), while there was no difference between before IAPS and after IAPS. More specifically, Tense Arousal was significantly higher in the High Arousal group than in the Low Arousal group after IAPS (*p* = 0.001), and the difference between the two groups after crime was marginally significant (*p* = 0.095).

### Behavioral Data

In the following analysis of the data obtained in the CIT, the target stimulus was excluded because it was different from the other stimuli (i.e., the probe and irrelevant stimuli), in that participants pressed different buttons in the CIT, and the focus of this study was not on the target but on the differences between the probe and irrelevant stimuli (see Matsuda et al., 2013a, for a similar analysis).

The mean RT were 456.89 (*SD* = 97.13) ms to the probe and 430.31 (*SD* = 79.23) ms to the irrelevant in the High Arousal group, and were 449.71 (*SD* = 98.93) ms to the probe and 434.88 (*SD* = 90.19) ms to the irrelevant stimuli in the Low Arousal group. A 2 (GROUP: High, Low) × 2 (STIMULUS TYPE: Probe, Irrelevant) repeated measures ANOVA was conducted, revealing a significant main effect of STIMULUS TYPE [*F*(1,19) = 11.279, *p* = 0.003, $\eta_{p}^{2}$ = 0.373]. The multiple comparisons analysis showed that mean RTs were significantly slower in response to the probe than the irrelevant stimuli (*p* = 0.003). There was no significant GROUP × STIMULUS TYPE interaction [*F*(1,19) = 0.910, *p* = 0.352, $\eta_{p}^{2}$ = 0.046].

### ERP Data

**FIGURE 3:**
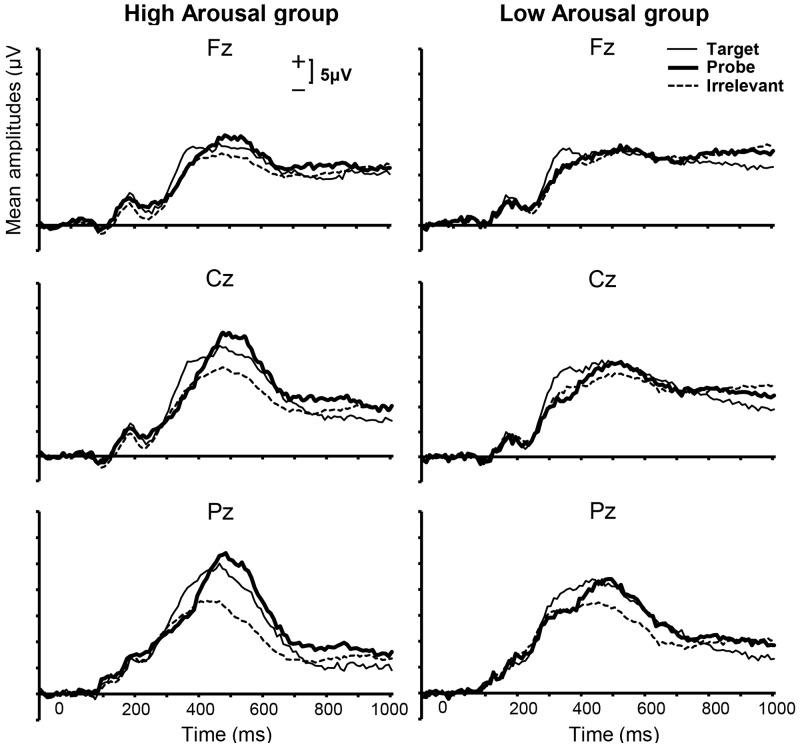
Grand-averaged ERPs for each group from 100 ms before to 1000 ms after stimulus presentation for midline electrodes and three stimulus categories. The time window from 300 to 600 ms was applied to calculate the positive peak.

Grand-averaged ERPs within categories of Stimulus Type for each group are shown in **Figure 3**. Visual inspection revealed prominent positive waves for both the High and Low Arousal groups. Because this component was parietally maximal and positive, and appeared with a peak at approximately 400 ms, we assumed that the component was the P300. Some previous studies have investigated the P300 at Pz using peak–peak (p–p) method, which computes the difference between the P300 peak and bottom peak from the P300 latency to approximately 1300 ms after stimulus onset, and emphasized the effectiveness of the P300-based CIT (e.g., Soskins et al., 2001; Rosenfeld and Labkovsky, 2010). However, in the present study we used the peak-amplitude method, because the clear negative peaks that are usually obtained following P300 peaks in P300-based CIT studies were not observed. This finding might be explained by previous reports that late positive potentials can be modulated in affective picture processing and motivated attentional processing (Schupp et al., 2000; Matsuda and Nittono, 2015). In addition, we did not limit our analysis to Pz because we focused on not only the difference in the P300 peak amplitude between probe and irrelevant stimuli but also the effect of emotional arousal between groups. Therefore, we calculated the largest positive peak in the range of 300–600 ms.

#### P300 Amplitude

**FIGURE 4:**
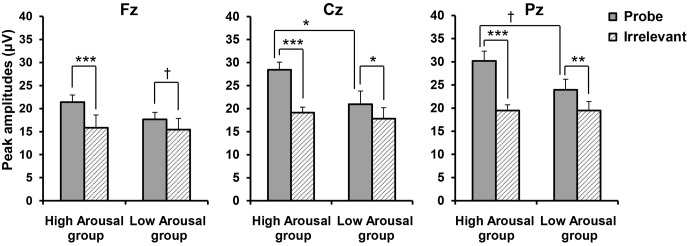
Peak amplitudes according to Stimulus Type, by group. Error bars indicate standard errors. ^†^*p* < 0.10, ^∗^*p* < 0.05, ^∗∗^*p* < 0.01, ^∗∗∗^*p* < 0.001.

The peak amplitudes according to Stimulus Type at each site for each group are shown in **Figure 4**. A three-way ANOVA compared amplitudes for GROUP (High, Low) × STIMULUS TYPE (Probe, Irrelevant) × SITE (Fz, Cz, Pz). Consistent with our hypothesis, we found a significant main effect of STIMULUS TYPE [*F*(1,19) = 53.962, *p* < 0.001, $\eta_{p}^{2}$ = 0.740] and a significant GROUP × STIMULUS TYPE interaction [*F*(1,19) = 10.705, *p* = 0.004, = 0.360]. We performed *post hoc* comparisons of a GROUP × STIMULUS TYPE interaction, but this only revealed a significant difference between P300 amplitudes in response to the probe and those in response to the irrelevant stimuli (*p* < 0.001), regardless of the emotional arousal group. Because we also found a significant main effect of SITE [*F*(2,38) = 24.495, *p* < 0.001, 𝜀 = 0.680, $\eta_{p}^{2}$ = 0.563], a significant STIMULUS TYPE × SITE interaction [*F*(2,38) = 21.015, *p* < 0.001, 𝜀 = 0.718, $\eta_{p}^{2}$ = 0.525], and a significant GROUP × STIMULUS TYPE × SITE interaction [*F*(2,38) = 4.217, *p* = 0.037, 𝜀 = 0.718, = 0.182], site appeared to significantly influence the differences between groups for each stimulus type. Thus, we performed *post hoc* comparisons including site (i.e., *post hoc* comparisons of the GROUP × STIMULUS TYPE × SITE interaction). As shown in **Figure 4**, it was revealed that in the High Arousal group, the amplitudes in response to the probe were significantly larger than those to irrelevant stimuli at all levels of site (Fz; *p* < 0.001, Cz; *p* < 0.001, Pz; *p* < 0.001), while in the Low Arousal group there were significant differences between the probe and irrelevant stimuli at Cz and Pz (Cz; *p* = 0.016, Pz; *p* = 0.002) and a marginally significant difference at Fz (*p* = 0.068). In addition, the amplitudes in response to the probe in the High Arousal group were significantly larger than those to the probe in the Low Arousal group at Cz (*p* = 0.039), although the amplitudes in response to the irrelevant stimuli were not different between groups. At Pz, the difference in responses to the probe between groups was marginally significant (*p* = 0.060). These results indicated that the findings of P300 amplitudes at Cz were consistent with our hypothesis.

Because detection efficiency is the main consideration in CIT studies, we calculated the difference score between the probe and irrelevant stimuli to reveal the detection efficiency in each group. The scores were 5.59 (*SD* = 1.38) μV at Fz, 9.31 (*SD* = 1.55) μV at Cz, and 10.72 (*SD* = 1.71) μV at Pz in the High Arousal group, and were 2.08 (*SD* = 1.09) μV at Fz, 2.95 (*SD* = 0.91) μV at Cz, and 4.62 (*SD* = 0.79) μV at Pz in the Low Arousal group. A two-way ANOVA compared the difference scores with GROUP (High, Low) × SITE (Fz, Cz, and Pz). We found a significant main effect of GROUP [*F*(1,19) = 10.705, *p* = 0.004, $\eta_{p}^{2}$ = 0.360] and SITE [*F*(2,38) = 21.015, *p* < 0.001, 𝜀 = 0.718, $\eta_{p}^{2}$ = 0.525], and a significant GROUP × SITE interaction [*F*(2,38) = 4.217, *p* = 0.037, 𝜀 = 0.718, $\eta_{p}^{2}$ = 0.182]. *Post hoc* comparisons showed that difference scores in the High Arousal group were significantly larger than those in the Low Arousal group at Cz and Pz (Cz; *p* = 0.002, Pz; *p* = 0.003), and marginally so at Fz (*p* = 0.061).

## Discussion

The main objective of the present study was to investigate whether emotional arousal at encoding influences the CIT. For this purpose, we manipulated the magnitude of emotional arousal before a mock crime, and examined the effects of this manipulation on the CIT using P300 amplitudes. Consistent with our hypothesis, P300 amplitudes in response to the probe stimulus were larger in the High Arousal group compared with the Low Arousal group, while amplitudes in response to the irrelevant stimuli were not different between groups. There were also significant differences in P300 amplitude between the probe and irrelevant stimuli in both groups.

The P300 amplitude differences we observed between the probe and the irrelevant stimuli by arousal group suggest that emotional arousal was a modifying factor that enlarged the probe-vs.-irrelevant difference and enhanced the detection efficiency of the CIT. The fact that we detected clear differences in P300 amplitude between the probe and irrelevant stimuli even in the Low Arousal group supports the notion that emotional arousal was not an essential factor for producing differences in responses to the probe and irrelevant stimuli. The current finding is similar to the results of previous memory studies that examined the interaction of the ERP old/new effect with arousal (Van Strien et al., 2009; Xu et al., 2015). Xu et al. (2015) reported that the old/new differences for high-arousing pictures were larger than those for low-arousing pictures at the retrieval stage. They also found greater positivity for high-arousing pictures at the encoding stage. They suggested that this positivity reflected stronger encoding activity causing more accurate discrimination of old items compared with new items. Emotional arousal in the High Arousal group may help strengthen encoding of memories and make the probe stand out more among the CIT stimuli. As a result, emotional arousal might lead to enhancement of both P300 amplitude and detection efficiency.

This emotional arousal effect is also similar to the emotional effects obtained in numerous affective ERP studies using emotionally arousing pictures (for a review, see Olofsson et al., 2008). According to these studies, arousing pictures generally elicit larger P300 amplitudes than neutral or low-arousing pictures (Johnston and Wang, 1991; Mini et al., 1996). The current results appear to replicate these studies. However, several potentially confounding factors prevent a full explanation. First, almost all of these studies focused on affective processing, not its interaction with memory processing. Few ERP studies have investigated the effect of arousal on memory processing, especially at the retrieval stage. Although some previously mentioned studies (Van Strien et al., 2009; Xu et al., 2015) measured ERPs at the retrieval stage, they did not report whether there were differences in ERP amplitudes between old high-arousing items and old low-arousing items. Palomba et al. (1997) and Dolcos and Cabeza (2002) reported an arousal effect and subsequent memory effect, but ERPs were measured only at the encoding stage, not the retrieval stage. Second, the stimuli used in the current study were not emotionally arousing pictures; the stimuli themselves were pictures of sharp tools, so were originally neutral stimuli would not be expected to elicit emotional arousal on their own. Although it is possible that emotional arousal at memory encoding could influence these originally neutral stimuli through participants’ experience with the mock crime, causing the pictures to function like emotionally arousing stimuli, it is unclear how emotional arousal enhances P300 amplitudes in the CIT.

One possibility is that emotional arousal at memory encoding enhances the encoding of the associated item so that the stimuli can be remembered well, and it results in inducing the stimulus significance. According to some neuroimaging studies, amygdala activity assessed by positron emission tomography (PET) imaging is stronger during memory encoding of emotionally arousing stimuli than neutral stimuli, and this activation is significantly correlated with individual subjects’ recognition memory enhancement (Hamann et al., 1999). In addition, one study reported that the amygdala activation was highly correlated with participants’ recall of these emotional arousing stimuli (Cahill et al., 1996). Several functional magnetic resonance imaging (fMRI) studies also confirmed the memory-enhancing effect of emotion, reporting that encoding of emotional pictures was associated with amygdala activation (Dolcos et al., 2003, 2004). These studies suggest that emotional arousal robustly activates the amygdala and enhances encoding processing. Seymour and Fraynt (2009) focused on encoding quality and investigated its effect in a CIT study. They reported that elaborately encoded probes afforded higher detection accuracy than poorly encoded information. Taken together, these findings suggest that emotional arousal at encoding may serve to enhance encoding processing through activation of the amygdala, as indicated by the neuroimaging studies mentioned above, and this high-quality encoding might cause increased P300 amplitudes in response to the probe in the High Arousal group, as indicated by Seymour and Fraynt (2009). Previous CIT studies have suggested that emotional factors (e.g., emotions related to deception, fear, and motivation to avoid detection) can increase the degree of stimulus significance or stimulus salience of the probe (Elaad and Ben-Shakhar, 1989; Verschuere and Ben-Shakhar, 2011). Unfortunately, significance ratings were not measured in this experiment and the relationship between memory and stimulus significance is still unclear. However, emotional arousal may strengthen encoding processing and also increase stimulus significance, potentially resulting in greater difference scores in a similar way.

A second possibility is that emotional arousal at memory encoding helps not only the encoding process, but aids retrieval of the emotional event. Previous neuroimaging studies have reported that amygdala activation is associated with the emotional retrieval process (e.g., Dolan et al., 2000; Smith et al., 2006), suggesting that the amygdala responds to and processes emotional information retrieved from hippocampus-dependent memory. It is possible that in the current study, the enhanced P300 in response to the probe in the High Arousal group reflected emotional arousal elicited at the retrieval process. However, it is difficult to isolate the effects on encoding from those on retrieval. To clarify the mechanisms involved in emotional arousal in the CIT, the subliminal presentation method is a useful tool to exclude the influence of conscious retrieval. Osugi and Ohira (2017) investigated the effect of emotional arousal at memory encoding using the subliminal presentation method, and reported that the difference scores on P300 amplitude were significantly greater in the High Arousal group than in the Low Arousal group, under both supraliminal and subliminal conditions. This finding supports the notion that P300 is derived from stimulus-driven processing, that is, without a retrieval process, and that emotional arousal may increase the significance of the stimulus at encoding. However, they suggested another possible explanation related to the dual route model proposed by LeDoux (1996), in which they speculated that only a probe encoded with high emotional arousal may be quickly processed via the Low road (a “quick and dirty” subcortical pathway for transferring rapid activity directly to the amygdala), independent of the top-down cortical loop, as threatening or fear-inducing stimuli are processed automatically. Emotional arousal at encoding may affect not only enhancement of the significance of the probe but also autonomic processing in the CIT. Further research will clarify this possibility.

Whereas P300 amplitudes were affected by emotional arousal and its detection efficiency was larger in the High Arousal group than in the Low Arousal group, RTs showed a CIT effect only and we did not find any effect of emotional arousal on RTs. The finding that mean RTs were significantly slower in response to the probe than the irrelevant stimuli is consistent with previous P300-based CIT studies (e.g., Farwell and Donchin, 1991) and RT-based CIT studies (e.g., Verschuere et al., 2010). Among studies using arousing stimuli, Van Strien et al. (2009) reported that responses to the high arousal pictures were slower than those to the low arousal pictures. However, this is the opposite result to that reported by Hofmann et al. (2009). The current study has a different protocol to these studies and there are few CIT studies focused on emotional arousal, as mentioned above. Thus, we believe that the RT results relating to emotional arousal remain controversial.

Regarding ERP components other than the P300, we did not obtain clear P200 or N200 components in grand average ERPs in either group. The P200 has been observed in several previous studies (e.g., Meixner and Rosenfeld, 2010; Hu et al., 2011), reporting larger P200 amplitudes in response to self-related information compared with other information. In addition, the N200 has also been reported in several CIT studies, and has been suggested to reflect a process of orienting attentional resources or response monitoring demands (e.g., Matsuda et al., 2009, 2013b; Gamer and Berti, 2010). Because Crowley and Colrain (2004) suggested in their review that an increase in the level of attentiveness of a subject produces a decrease in P200 amplitude, the lack of clear P200 peak in the current study might indicate increased attentiveness of participants. Several previous studies reported that N200 was absent during correct responses (Holroyd et al., 2008) and in responses to easily discriminable stimuli (Nieuwenhuis et al., 2004). In the current study, the stimuli used in the CIT were discriminable, and only epochs on correct trials were analyzed, potentially reducing the amplitude of the N200.

The current study included several limitations that should be considered, particularly regarding the number of participants. Although we found significant effects of emotional arousal, suggesting the absence of type II errors regardless of the small sample size, it should be noted that the small sample size limits the generalizability of our conclusions. Because of the potential non-representativeness of the sample, the possibility of type I errors caused by individual differences, such as gender, age, and nationality, should be considered. Examining this effect in more detail will require further research with a larger sample size. Moreover, it is possible that the effects of arousal in this study were exaggerated or distorted because of the small sample size. The reproducibility of our findings should be investigated in future replication studies.

Another limitation is related to our method for checking the manipulation of emotional arousal, which relied solely on a self-report measure. The results revealed that the subjective arousal levels were significantly changed by the manipulation and mock crime in this study, suggesting that the present results did not suffer from this limitation. However, it is possible that physiological arousal levels were changed by the manipulation and mock crime. To resolve this possibility in the future, it would be helpful to measure physiological responses during encoding.

In addition, it is possible that the interaction between the two arousal tasks (emotional arousal manipulation task and mock crime) also affected the current findings. Although we assumed that state arousal manipulated by affective pictures would not enhance the P300 solely without the mock crime procedure, state arousal may have influenced the P300 through the mock crime. Future studies using other methods of manipulation may clarify this potential interaction.

The manipulation of emotional arousal using arousing pictures may be seen to be unrealistic and not ecologically valid. In this study, arousing pictures were used only to induce emotional arousal before the mock crime and were not directly or contextually related to the CIT, as we used different stimuli in the CIT. However, the possibility that this manipulation influences the CIT somehow because it is unrealistic cannot be fully excluded. Further research using different manipulations would help to clarify this point.

In addition, although we assumed that the effects of emotional arousal shown in this study were not due to the characteristics of the mock crime, it is possible that the type of mock crime influenced the effects of emotional arousal. Because a new type of mock crime was applied in this study, it would be helpful to investigate the effect using different types of mock crime in future research.

Finally, it remains unclear whether emotional arousal effects are accurately reflected by the autonomic nervous system (ANS) measurements often used in CIT studies. Because the P300 and ANS measurements are based on different detection theories in the CIT in recent CIT studies (Selle et al., 2016, Klein Selle et al., 2017; Rosenfeld et al., 2017), we cannot directly generalize the effects of emotional arousal with ANS measurements from the current findings. Although it is possible that the effects of emotional arousal were reflected only on the P300, it is also possible that the effects would be shown with ANS measurements using the same procedures in this study. Future studies using various ANS measurements should be conducted to investigate these possibilities.

To our knowledge, the present study was the first attempt to investigate the influence of emotional arousal on the CIT using the P300, and the first empirical demonstration of the effect of emotional arousal on memory encoding in the CIT. The current findings support the notion that emotional arousal plays a key role in enhancing detection ability in the CIT, and also help clarify the external validity of the CIT studies. Although many CIT researchers have expressed concern about the difference between arousal in the laboratory setting and in practical applications, our results confirm the connection between the two, demonstrating that we can investigate and detect related information in the CIT even when the test is conducted in a low-arousal setting in the laboratory.

## Author Contributions

AO designed the study, contributed to data collection and analysis, and wrote the initial draft of the manuscript. HO contributed to interpretation of data, and assisted in the preparation of the manuscript. The final version of the manuscript was approved by all authors.

## Conflict of Interest Statement

The authors declare that the research was conducted in the absence of any commercial or financial relationships that could be construed as a potential conflict of interest.

## Footnotes

1 The IAPS slide numbers were as follows: high emotionally arousing picture, 3005.1, 3400, 5972, 6260, 6550, 6831, 9300, 9600, 9635.1, and 9921; low emotionally arousing picture, 2205, 2276, 2455, 3300, 9000, 9220, 9280, 9331, 9340, and 9342.

2 Mean arousal, valence and dominance scores were as follows: high emotionally arousing pictures, 6.45, 2.42, and 3.29; low emotionally arousing pictures, 4.39, 2.58, and 4.01.
